# Loss of TINCR expression promotes proliferation, metastasis through activating EpCAM cleavage in colorectal cancer

**DOI:** 10.18632/oncotarget.8141

**Published:** 2016-03-17

**Authors:** Zuo-yang Zhang, Yan-xia Lu, Zhe-ying Zhang, Ya-ya Chang, Lin Zheng, Li Yuan, Fan Zhang, Yu-han Hu, Wen-juan Zhang, Xue-nong Li

**Affiliations:** ^1^ Department of Pathology, School of Basic Medical Sciences, Southern Medical University, Guangzhou 510515, China; ^2^ Department of Pathology, Guangzhou Women and Children's Medical Center, Guangzhou 510515, China

**Keywords:** TINCR, EpCAM, c-Myc, sp1, colorectal cancer

## Abstract

Long non-coding RNAs (lncRNAs) are involved in kinds of human diseases, including colorectal cancer (CRC). TINCR, a 3.7 kb long non coding RNA, was associated with cell differentiation in keratinocyte and gastric cancer cells. However, little is known about the role of TINCR in regulation CRC progression. Here, we showed that lncRNA TINCR was associated with CRC proliferation and metastasis. TINCR was statistically downregulated in CRC tissues and metastatic CRC cell lines compared with their counterparts. TINCR was reversely correlated with CRC progression and promoted tumor cells growth, metastasis *in vivo* and *in vitro*. While overexpression of TINCR had opposite effect. In addition, we also found that TINCR specifically bound to EpCAM through RNA IP and RNA pull down assays. Loss of TINCR promoted hydrolysis of EpCAM and then released EpICD, subsequently, activated the Wnt/β-catenin pathway. Further studies shown that c-Myc repressed the expression of TINCR through repressing sp1 transcriptive activity, which established a positive feedback loop controlling c-Myc and TINCR expression. These findings elucidate that loss of TINCR expression promotes proliferation and metastasis in CRC and it could be considered as a potential cancer suppressor gene.

## INTRODUCTION

Colorectal cancer (CRC) is the third most commonly diagnosed cancer in males and the second in females worldwide [1]. The estimated 5-year survival rate range from 90% for stage I patients to 10% in the metastatic cases despite recent therapeutic advances in CRC treatment [2, 3]. Recognized predictive markers for metastases are emergency presentation.

LncRNAs, a subgroup of non-protein coding transcripts, ranging in length from 200 bp to tens of kilobases (kb), and accumulating evidences have shown that lncRNAs may promote the formation and progression of colorectal cancer [4–6]. Firstly, lncRNAs can increase the stability of chromatin. HOTAIR cooperating with Polycomb complex PRC2 could reprogram chromatin organization and promote cancer metastasis in breast cancer, and colorectal cancer [7]. CCAT2 is highly overexpressed in microsatellite-stable colorectal cancer, and promotes tumour growth, metastasis and chromosomal instability [8]. In addition, several lncRNAs function as transcriptional activators or repressors in CRC. LncRNA-CCAT1 promotes long-range chromatin interactions at the MYC locus and dramatically increases the expression of MYC [9]. The fact that overexpression of lncRNA-ROR in wild HCT116 cells could significantly decrease the expression of p53 indicates that ROR is a strong repressor of p53 [10]. Furthermore, the ability of lncRNA to interact with signal transduction pathways allows them to regulate the function of cancer cells. Colorectal cancer-associated lncRNA (CCAL) promotes CRC progression by targeting AP-2α, and then activated the Wnt/β-catenin pathway [11]. LncRNA-PVT-1 decrease the proliferation and invasion capabilities by acting the TGF-β signal pathway and the apoptotic signals in CRC cells [12].

Terminal differentiation-induced lncRNA (TINCR) is a 3.7 kb lncRNA which is isolated from human well differentiated somatic tissues. TINCR interacts with a range of differentiation mRNAs though a specific motif. TINCR also binds to the stau1 protein and mediates the stabilization of differentiation mRNAs [13, 14]. TINCR may be significantly downregulated in low differentiated human squamous cell cancer species. Morphologically, colorectal cancer could be categorized as high, middle, and poor differentiated groups. Nevertheless, the potential roles and biological mechanisms of TINCR in the progression of CRC remain largely unknown.

In this study, we demonstrated that lncRNA-TINCR which associated with tissues differentiation, was downregulated in the colorectal cancer tissues and inhibited CRC cell proliferation, invasion and metastasis. TINCR specifically bind to EpCAM, and loss of TINCR accelerating the hydrolysis of EpCAM and released EpICD, then activate the EpICD-Wnt/β-catenin signaling in CRC. We also revealed that c-Myc could repress the expression of TINCR by a sp1-dependent way. This study provides a novel mechanism for TINCR in proliferation, migration, and metastasis in CRC.

## RESULTS

### Downregulation of LncRNA-TINCR correlated with CRC progression

**Figure 1:**
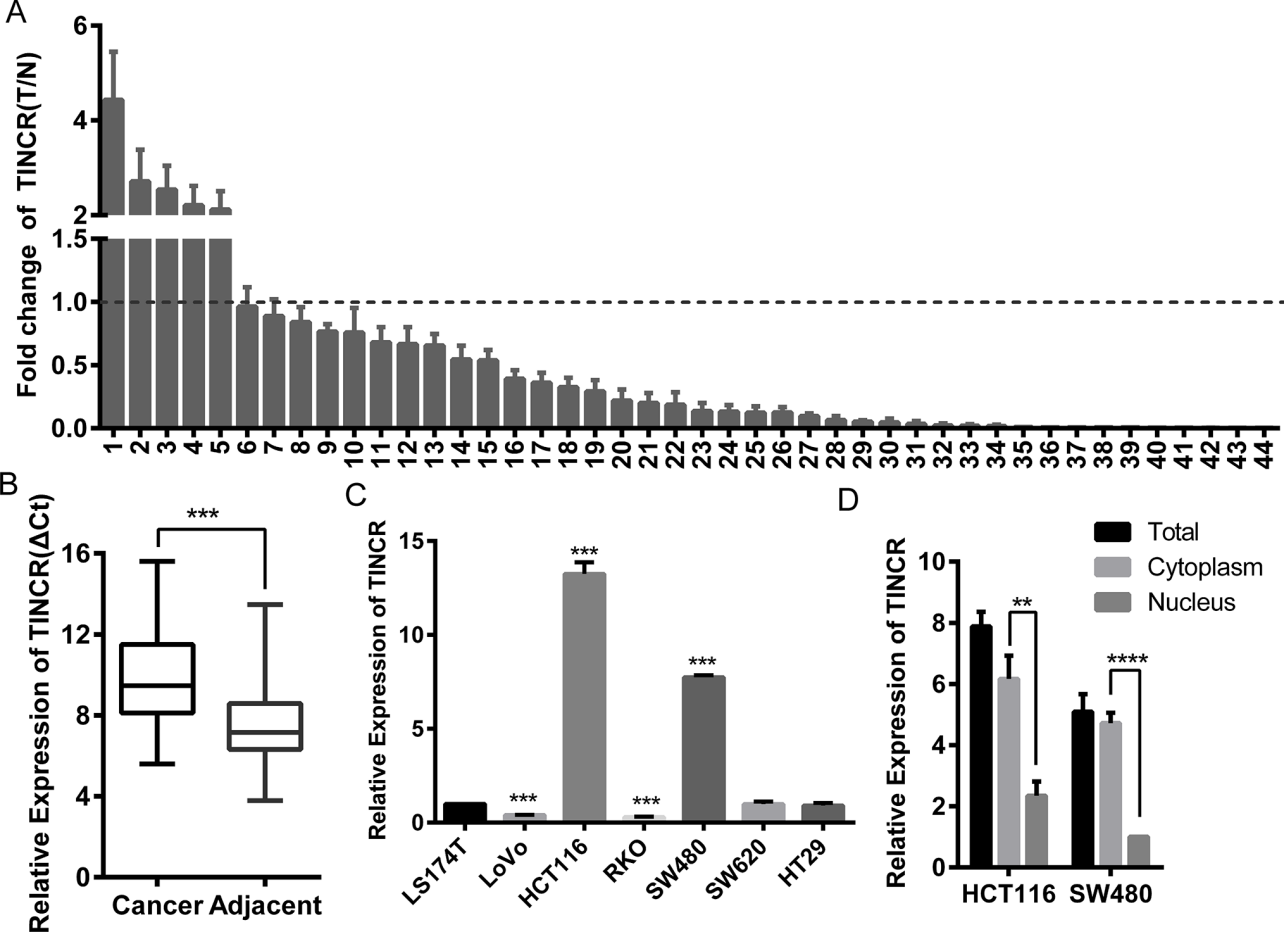
Downregulation of LncRNA-TINCR correlated with CRC progression (**A**) qRT-PCR analysis of lnc-TINCR expression in 44 paired colorectal cancer tissues; Shown data are mean ± SD from 3 independent experiment. TINCR was quantified relative to the matched adjacent no tumor tissues. (**B**) Comparison of TINCR expression in 44 paired CRC tissues with adjacent tissues. Shown data are mean ± SD from 3 independent experiment. (**C**) qRT-PCR was used to analysis TINCR expression in CRC cell lines. Shown data are mean ± SD from 3 independent experiment. (**D**) qRT-PCR analysis of TINCR in HCT116 and SW480 total cell, cytoplasmic and nucleus respectively. Shown data are mean ± SD from 3 independent experiment.

We relied on Oncomine, a cancer microarray database and web-based data-mining platform [15, 16], to identify the expression level of TINCR in colorectal cancer tissues, and the result indicated that TINCR was significantly downregulated in colorectal cancer tissues comparing with adjacent tissues. (Supplementary, Figure S1A, S1B). We also analyzed the expression of TINCR in tumour and paired non-tumour tissues obtained from 44 patients with CRC. The qRT-PCR analysis revealed significantly lower TINCR expression in 38 of 44 CRC specimens (*p* < 0.0001) (Figure 1A and 1B).

We next examined the relationship between TINCR expression levels and the clinicopathological characteristics of the tumour tissue samples. As shown in Supplementary Table 4, the TINCR expression level was reversely correlated to serosal invasion (*p* = 0.001), lymph metastasis (*p* = 0.037), and tumour node metastasis (TNM) classification (*p* = 0.016), while positively correlated with differentiation degree (*p* = 0.017). Moreover, the expression level of TINCR in the high malignant potential cell lines LoVo, RKO, and SW620 was significantly decreased compared with low malignant potential cell lines HCT116, SW480 and LS174T (Figure 1C). The transcript for TINCR was mainly located in the cytoplasm of HCT116 and SW480 cells by separating nuclear and cytoplasmic RNA fraction (Figure 1D). These results suggest that the decreased TINCR expression is clinically relevant to the metastasis of CRC.

### TINCR inhibits CRC cells proliferation, migration and metastasis

**Figure 2:**
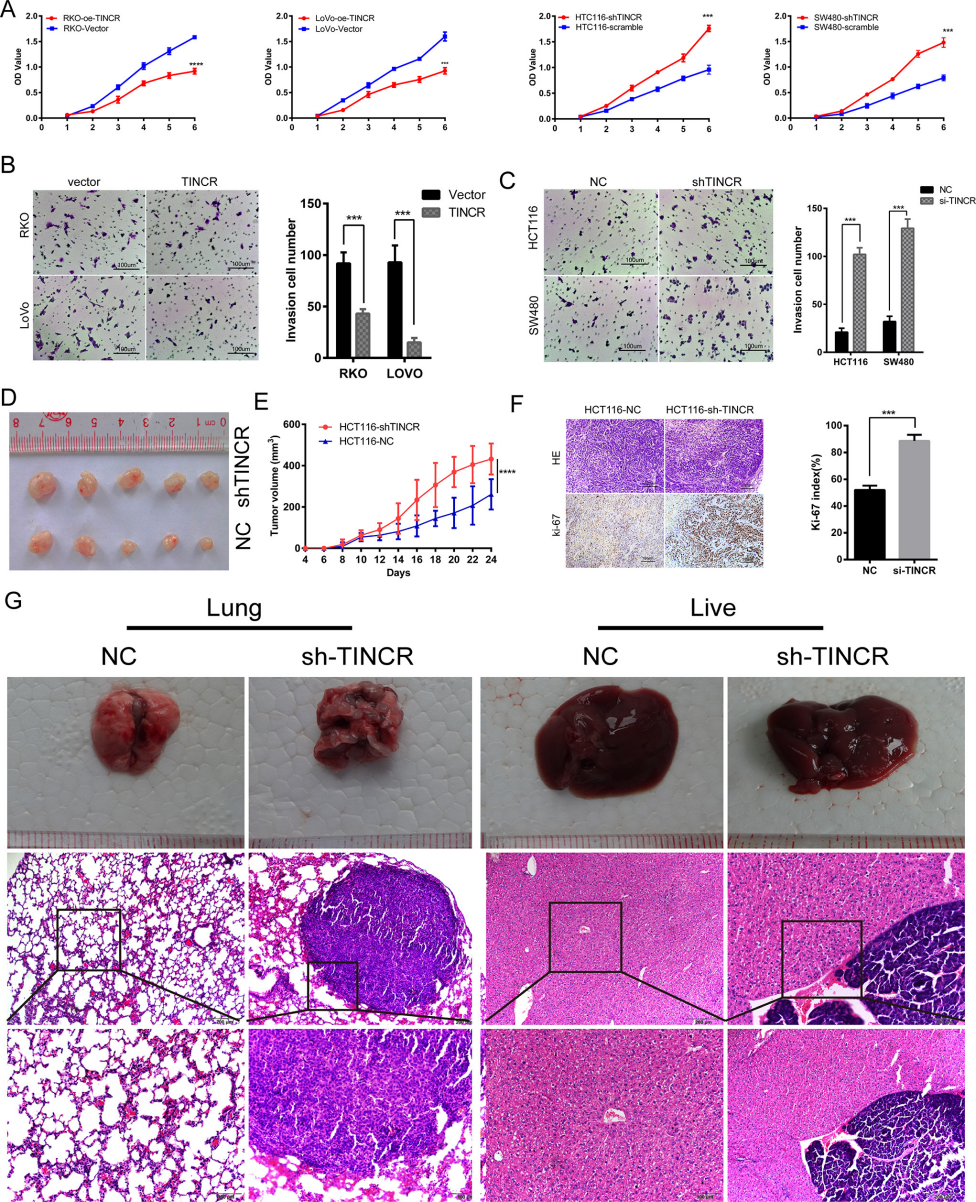
TINCR inhibits CRC cells proliferation, migration and metastasis (**A**) Effects of oe-TINCR and sh-TINCR on cell proliferation were determined by CCK8 cell proliferation assay. Shown data are mean ± SD from 3 independent experiment. (**B**, **C**) The migration capacity was determined by colony formation assay after oe-TINCR (B) or sh-TINCR (C). Data shown are mean ± SD of 3 independent experiments. (**D**) Effects of HCT116 cell line sh-TINCR on subcutaneous tumor generation (*n* = 5). (**E**) Tumor sizes were measured on the indicated days. Data, tumor volume mean ± SD. (**F**) Ki-67 index was analyzed to evaluate the capacity of proliferation in subcutaneous tumor cells. HE (up) and IHC images (down), The Ki-67 index was calculated as the number of Ki-67 positive cells divided by the number of total cells × 100%. (**G**) Images of the hepatic and lung metastases, and the HE stain of the metastatic livers and lungs of the mice in the tail vein injected metastasis assay (*n* = 5 per group).

To gain insight into the function of TINCR in CRC progression, TINCR was overexpressed or silenced in CRC cell lines (Supplementary Figure S2A, S2B). Stable ectopic expression of TINCR decreased not only proliferation (*p* < 0.001, Figure 2A, Supplementary Figure S2C) but also the migration (Figure 2B, Supplementary Figure S2D) of RKO and LoVo cells. In contrast, knockdown of TINCR in HCT116 and SW480 cells had the opposite effects (*p* = 0.001. Figure 2A, 2C and Supplementary Figure S2E).

Consistent with *in vitro* observations, xenograft tumours formed in sh-TINCR group were generally larger than those in the control group (Figure 2D). Tumours growth in the sh-TINCR group was significantly more rapid than that in the control group (Figure 2E). Additionally, the tumours developed from sh-TINCR cells displayed higher ki-67 proliferation index (Figure 2F). All (5/5) of the mice in HCT116-shTINCR group displayed metastatic foci in the lung and 4/5 of these mice had metastasis foci in the liver. However, only 1/5 of mice in HCT116-NC group had lung metastasis foci and no mice in HCT116-NC group develop hepatic metastasis foci (Figure 2G). We also counted the number of metastatic nodules in each liver and lung, and found that TINCR knockdown increased the number of metastatic nodules compared to the control cells (lung: 4:1; live: 2:0). Collectively, these results indicate that loss of TINCR expression profoundly promotes tumour growth and metastasis.

### TINCR induces cell cycle arrest and apoptosis

**Figure 3:**
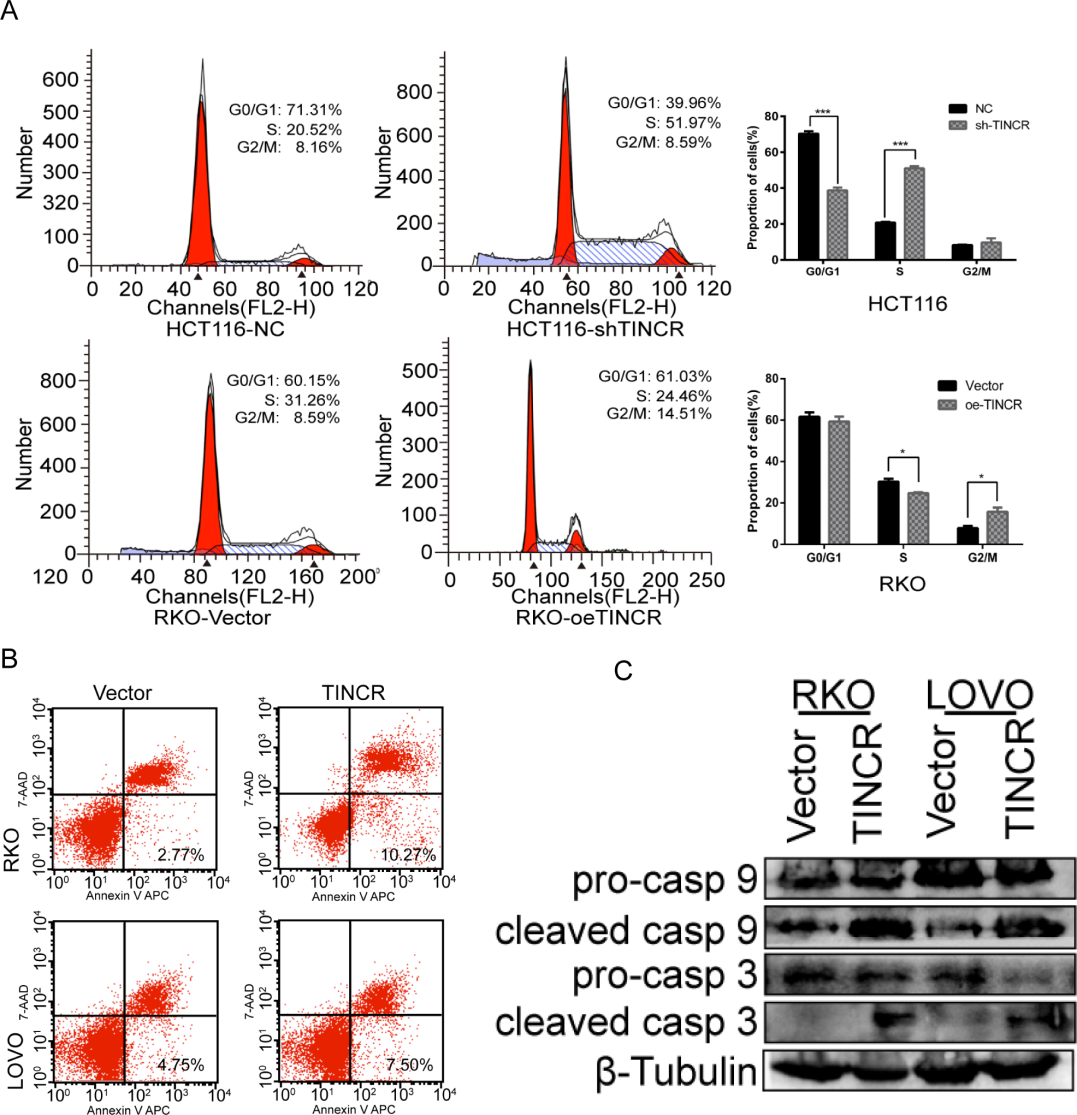
TINCR induces cell cycle arrest and apoptosis (**A**) Cell cycle arrest after oe-TINCR or sh-TINCR measured by flow cytometry. Shown data are mean ± SD from 3 independent experiment. (**B**) Overexpression of TINCR promotes apoptosis as analyzed by flow cytometry. Shown data are mean ± SD from 3 independent experiment. (**C**) Western blotting analyze the expression of apoptosis associated proteins caspase 3 and caspase 9 after oe-TINCR.

We addressed that whether the deceleration of proliferation might be a result of cell cycle arrest or cell apoptosis inducing. The downregulation of TINCR in HCT116 cells significantly decreased the proportion of cells in G0/G1 phase (from 71.31% ± 1.57 to 39.96% ± 1.21), while increased the proportion of cells in S phase (from 20.52% ± 0.97 to 51.97% ± 1.68). The overexpression of TINCR drove progression beyond the G2/M transition in RKO cell lines. The percentage of cells in G2/M phase in RKO/oe-TINCR (14.51% ± 1.35) was significantly higher than that in RKO/Vector cells (8.59% ± 0.87) (Figure 3A). The upregulation of TINCR induced a significant increase of early apoptosis in RKO cells (2.77% ± 1.05 to 10.27% ± 1.29) and LoVo cells (4.75% ± 0.82 to 7.50% ± 1.33) (Figure 3B). Furthermore, the expression levels of some of apoptosis associated proteins were detected. The upregulation of TINCR activated caspase 3 and caspase 9 in RKO and LoVo cells (Figure 3C). These results indicated that the overexpression of TINCR induces cell cycle arrest and apoptosis in CRC cells, which contributes to the growth inhibition properties of TINCR.

### TINCR specifically binds to EpCAM and regulates its proteolysis

**Figure 4:**
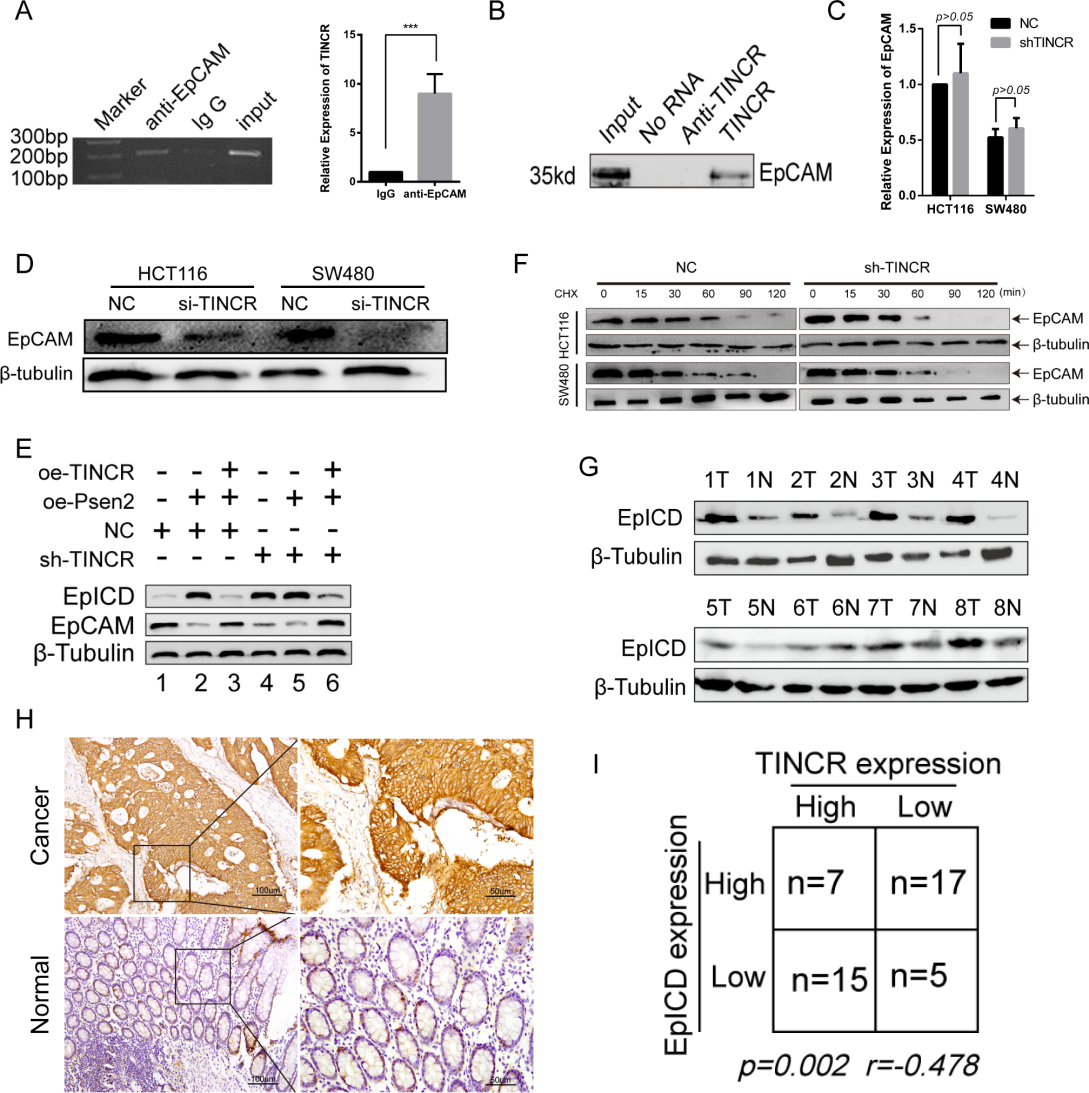
TINCR specifically binds to EpCAM and regulates its proteolysis (**A**, **B**) RNA IP assay (A) and RNA pull down (B) assay were used to evaluate the bind of TINCR and EpCAM. Shown data are mean ± SD from 3 independent experiment. (**C**, **D**) qPCR and western blotting analysis the EpCAM mRNA (C) and protein (D) expression in HCT116/sh-TINCR and SW480/sh-TINCR cell lines. Data shown are mean ± SD from 3 independent experiment. (**E**) Western blotting analysis the effect of oe-TINCR on the proteolysis function of PSEN2 towards the EpCAM in HCT116 cell line. (**F**) Effect of sh-TINCR on the half-time of EpCAM in HCT116 and SW480 cell lines. Cells were treated with cycloheximide (CHX, 20 mg/ml) for the indicated periods of time. (**G**) Western blotting analysis of EpICD in 8 paired CRC tissues and their counterpart normal tissues. (**H**) IHC evaluated the expression level of EpICD in 44 CRC tissues and associated normal colorectal tissues. (**I**) Spearman correlation analyses the relationship between TINCR and EpICD.

EpCAM was one of the disrupted genes when TINCR was depleted and had the highest score by transcript profiling [13]. RNA immunoprecipitation (RIP) assays demonstrated that EpCAM Ab precipitated the lncRNA-TINCR while the IgG Ab could not (Figure 4A). Secondary, biotin RNA pull-down assay was performed, suggesting that TINCR directly interacted with EpCAM (Figure 4B, Supplementary Figure S3). Knockdown of TINCR did not change the EpCAM mRNA but downregulated the EpCAM protein levels in CRC cells (Figure 4C and 4D). EpCAM would be hydrolyzed and consecutively release EpICD (EpCAM c-term, intracellular domain) when catalyzed by psen2 [17]. The downregulation of TINCR increased the expression of EpICD (Figure 4E, line1, line4). The overexpression of psen2 alone or in HCT116/sh-TINCR cells upregulated the expression of EpICD and reduced the expression of EpCAM (Figure 4E, line2, line4; Supplementary Figure S4). However, all these effects disappeared when TINCR was re-expressed in HCT116 NC or sh-TINCR cells (Figure 4E, line2, 3; line5, 6). Moreover, the knockdown of TINCR led to a robust decreased in EpCAM protein half-life in HCT116 and SW480 cells (Figure 4F). Considering the intracytoplasmic location of TINCR, these results indicate that the inhibition of TINCR may accelerate the cleavage of EpCAM and release EpICD.

As shown in Figure 4G, EpICD was upregulated in the CRC tissues compared with their normal mucous. We next analyzed the expression of EpICD in the same 44 paired CRC tissues by IHC (Figure 4H). Spearman correlation analyses showed that TINCR and EpICD were inversely related in expression (*p* = 0.005, *r* = −0.478, Figure 4I).

### Downregulation of TINCR activates WNT/β-catenin pathway in CRC

**Figure 5:**
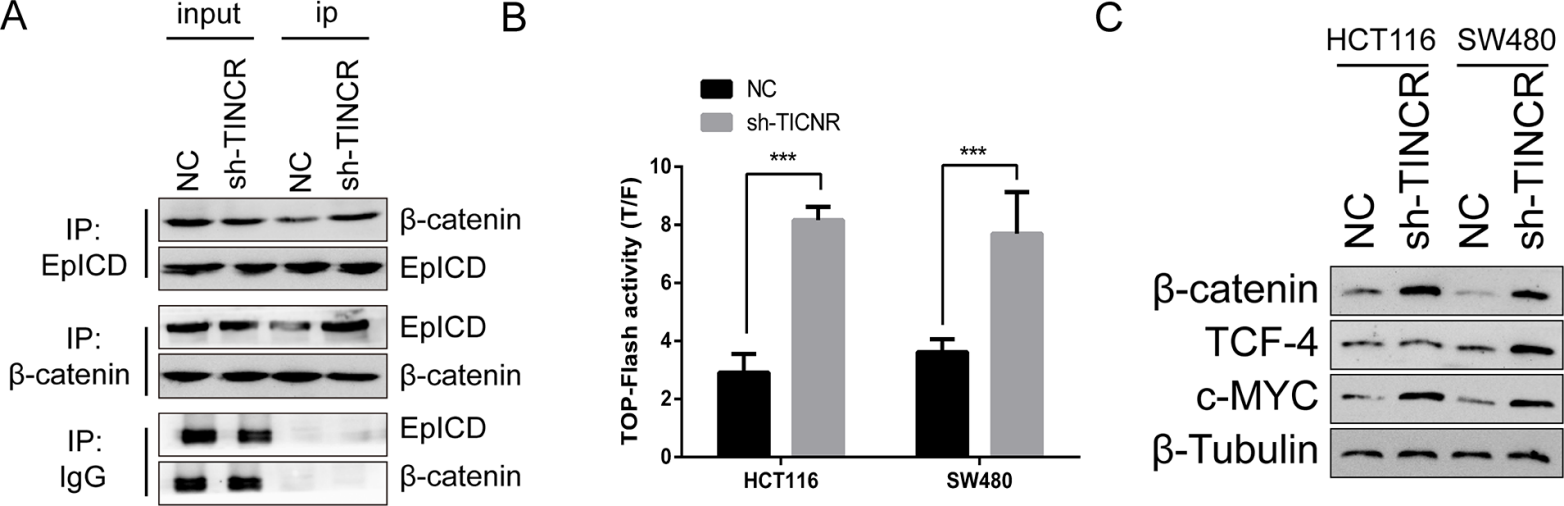
Downregulation of TINCR activates WNT/β-catenin pathway in CRC (**A**) Co-IP assay indicate that sh-TINCR promote the formation of EpICD and β-catenin complex in HCT116 cell. (**B**) TOP-FOP flash assay was performed to evaluate the effect of sh-TINCR on the wnt/β-catenin pathway. Shown data are mean ± SD from 3 independent experiment. (**C**) Western blot analysis the expression of wnt pathway associated genes.

EpICD is one of the components of Wnt pathway. EpICD colocalized with FHL2 and β-catenin to form a nuclear protein complex, leading to gene transcription [18]. Consistently, in HCT116 cells which showed the formation of EpICD and β-catenin complex (Figure 5A). The knockdown of TINCR resulted in 200%−300% increment of TOP-Flash reporter gene activity (Figure 5B). Moreover, the expression of β-catenin, as well as the Wnt target genes TCF4, and c-Myc were significantly induced in TINCR-knockdown cells (Figure 5C). In conclusion, the activation of WNT/β-catenin pathway is mediated by TINCR depletion in CRC.

### C-Myc represses the expression of TINCR through inhibiting the transcriptive activity of Sp1

**Figure 6:**
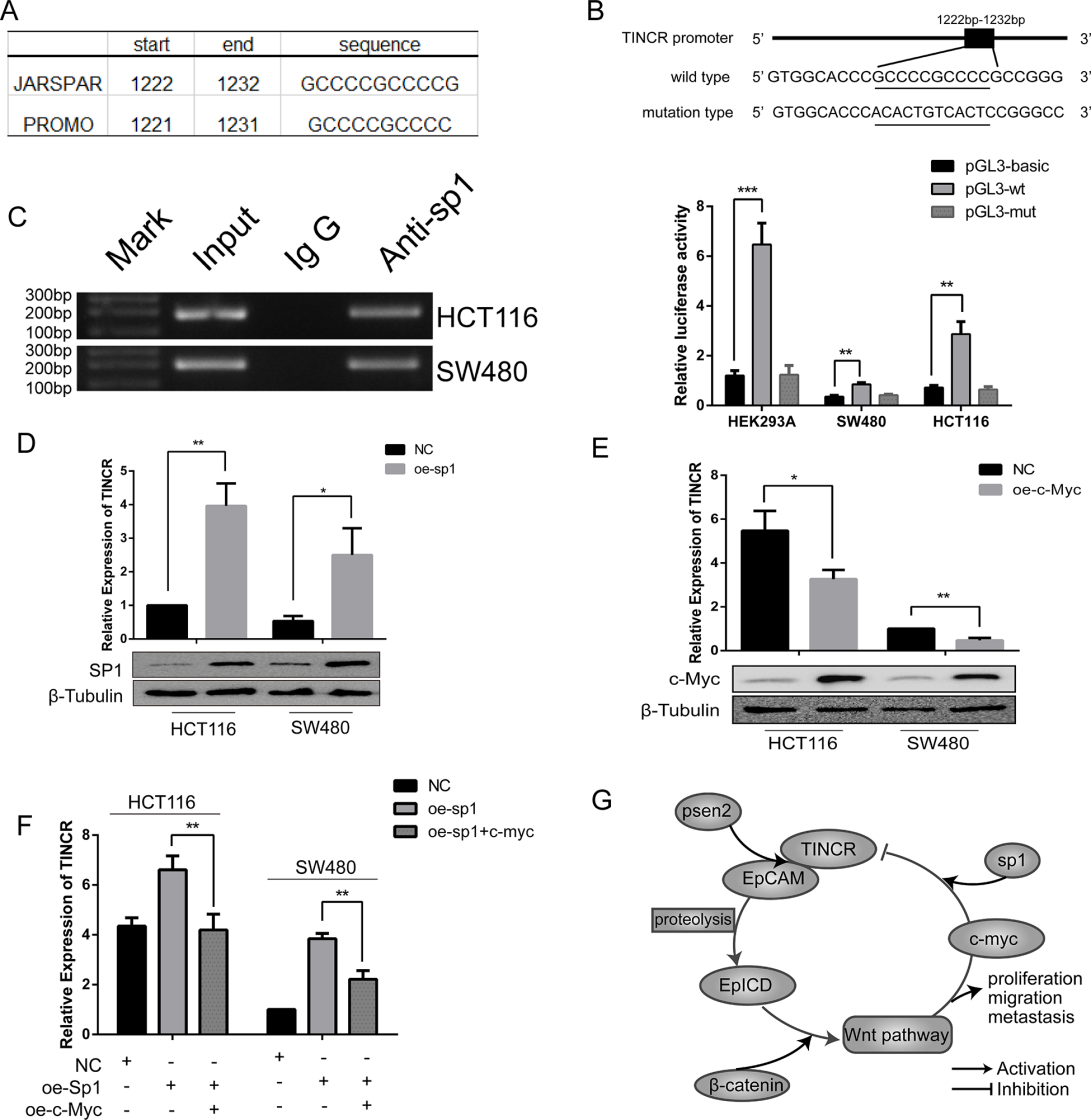
C-myc repress the expression of TINCR through inhibiting transcriptive activity of sp1 (**A**) Bioinformatics analysis the binding site of sp1. (**B**) Relative luciferase activity of the wt TINCR promoter or the mut promoter in the HEK292A, HCT116 and SW480 cells transfected sp1 plasmid or empty vectors. Shown data are mean ± SD from 3 independent experiment. (**C**) ChIP assay was used to confirm the binding of sp1 with the TINCR promoter. Shown data are mean ± SD from 3 independent experiment. (**D**) Overexpression of Sp1 in HCT116 and SW480 increase the expression of TINCR. Shown data are mean ± SD from 3 independent experiment. (**E**) Overexpression of c-Myc in HCT116 and SW480 increase the expression of TINCR. Shown data are mean ± SD from 3 independent experiment. (**F**) Relative expression of TINCR in HCT116 and SW480 cell lines which co-infected with sp1 and c-Myc. Shown data are mean ± SD from 3 independent experiment. (**G**) A proposed model of reciprocity between lnc-TINCR and EpCAM regulates proliferation, invasion and metastasis in colorectal cancer. Error bars, mean ± SD of 3 independent experiments.

Potential transcription factors (TFs) promoting the expression of TINCR were analyzed by bioinformatics algorithms. Sp1 was the predicted TF with one binding site on the TINCR promoter showed by the JARSPAR, and PROMO databases (Figure 6A and Supplementary Figure S5, S6). An obvious increasing Sp1-binding activity on the TINCR promoter was observed by the dual luciferase reporter assays (Figure 6B). Additionally, ChIP assays confirmed the Sp1 binding to TINCR promoter (Figure 6C). The overexpression of Sp1 significantly increases the expression of TINCR in HCT116 and SW480 cells (Figure 6D). It reported that c-Myc binding to the Sp1 transcription factor via the c-Myc central region and inhibition of Sp1 transcriptional activity on the cell cycle/growth arrest mechanism [19]. TINCR, transcribed by Sp1, was repressed by c-Myc (Figure 6E). Moreover, the increasing expression of TINCR by Sp1 was partly suppressed by c-Myc (Figure 6F). Collectively, TINCR induces cell cycle arrest and inhibits proliferation may be due to the suppression of c-Myc. On the other hand, these findings establish a positive feedback loop controlling c-Myc and TINCR expression (Figure 6G).

## DISCUSSION

Accumulating evidence indicates that the abnormal expression of lncRNAs is a frequent molecular event in human malignances including CRC [20, 21]. In this study, we ascertained that the expression of TINCR was downregulated in CRC tissues, and TINCR is significantly reversely associated with the invasion and metastasis of CRC as well as a more advanced TNM stage. Additionally, overexpression of TINCR could significantly inhibit CRC cell proliferation *in vitro* by causing cell cycle arrest and inducing apoptosis. However, TINCR is strongly upregulated in human gastric carcinoma (GC) and promoted cell growth [22]. The inconsistency of TINCR in regulating cell growth in CRC and GC may be due to the differential expression phenotype.

The molecular mechanisms of TINCR on cell proliferation remain poorly identified. It reported that lncRNAs could act together with specific proteins when performing its functions [23–24]. In the present work, we confirm that TINCR specifically binds to EpCAM and protects it from been cleaved, and loss of TINCR expression accelerates the hydrolysis of EpCAM and release EpICD. EpCAM, the epithelial cell adhesion molecular, is a 40 kd cell surface glycoprotein identified as a marker for epithelial cancers, as well as a marker for cancer stem cells and hESCs [25–27]. Targeted drugs catumaxomab and MT110 are specific antibodies of EpCAM [28]. Previous studies indicated that low expression of EpCAM correlated with a tumour-promoting role and poor patient survival in colorectal cancer [29, 30]. Taken together, the hydrolysis of EpCAM regulated by TINCR may implicate the therapeutic value of TINCR for CRC. We also confirmed the formation of EpICD and β-catenin complex and shown that the downregulation of TINCR promoted the expression of β-catenin, TCF4 and c-Myc. In other words, downregulation of TINCR activates Wnt/β-catenin pathway in CRC, which explored a novel mechanism for TINCR inhibited cell growth and metastasis.

Sp1 promotes a variety of cancer associated genes that are implicated in cell growth, differentiation, and apoptosis [31–33]. We confirmed that sp1 bound to the promoter of TINCR and slightly positively regulated the promoter activity. Moreover, c-Myc could repress the expression of TINCR by a sp1-dependent way. Together these studies provide a molecular model for c-Myc in suppressive regulation of TINCR promoter activity and expression. These results will help to better understand the role of TINCR in inducing cell cycle arrest to inhibit proliferation.

Taken together, our results indicate that TINCR is a tumour suppressor that, when downregulated, may promote CRC growth and metastasis, and accelerate hydrolysis of EpCAM. With regard to the fantastic potential applications of targeted drugs for EpCAM, this finding suggests the considerable therapeutic value of TINCR for CRC.

## MATERIALS AND METHODS

### Clinical specimens and cell lines

Total 44 human colorectal cancer tissues and their paired normal colorectal mucosa were obtained from Nanfang Hospital, Southern Medical University (Guangzhou, China). Patients and/or their relatives approved the use of these clinical materials for this research according to the Ethics Committee of Southern Medical University. All tissues were frozen in liquid nitrogen immediately after surgical exeresis, and then stored at −80°C. The CRC cell lines used in this research were obtained from ATCC, and cultured in RMPI-1640 medium containing 10% FBS in CO_2_ at 37°C according to the recommendation by ATCC.

### Plasmids construction and RNA interference

The full length of TINCR, psen2, c-Myc and Sp1 were amplified by PCR using cDNA from non-tumour colorectal mucosal tissues, and cloned into pcDNA3.0 vectors respectively. The pcDNA3.0-anti-TINCR was generated with the antisense of TINCR. The TINCR promoter DNA fragment containing the wild-type (wt) or mutant (mut) putative target site of the transcription factor Sp1 were subcloned into the pGL3-basic vectors. All the PCR primers were collected in Supplementary Table S1. shRNA targeting TINCR(5′-AATACCTGCTACTTCATGC-3′), and NC (5′-AACCTTACAATGAATCTAC-3′) were purchased from GeneChem, shanghai.

### RNA isolation, quantitative reverse transcription PCR, western blotting

RNA isolation, qRT-PCR and western blotting were performed as previously described [34–36]. The qRT-PCR primers and antibodies were listed in the Supplementary Table S2 and Table S3 respectively.

### Proliferation, plate colony formation, cell migration, and invasion assays, flow cytometry

The cell proliferation, plate colony formation, cell migration, invasion assays and Flow Cytometry were performed as previously described [37].

### Luciferase reporter assay

Cells were seeds in 24-wells plates (1 × 10^5^/well) and cultured for 24 hours. Then, either pGL3-wt promoter vectors or pGL3-mut promoter vectors were co-transfected with pcDNA3.0-sp1 plasmids in HCT116 and SW480 cells using lipofectamine 3000 (Invitrogen, USA). The control luciferase plasmids pRL-TK was also transfected. Luciferase activity was measured 48 hours after transfection according to the Dual-luciferase Reporter Assay System (Promega, catalogue E1910).

### Chromatin immunoprecipitation

Chromatin immunoprecipitation was preformed according to the ChIP assay kit (Millipore, catalogue EZ17-371). Briefly, 1 × 10^6^ cells were lysed by sodium dodecyl sulfate lysis buffer, and sheared to length between 200 bp to 1000 bp using sonication. Sp1 or IgG antibody was used to immunoprecipitation with protein-DNA complex, and then elution the DNA from the antibody. PCR was performed with primers specific for TINCR promoter (Supplementary Table S2).

### RNA immunoprecipitation

RNA immunoprecipitation (RIP) experiments were performed according to the instruction provided by the kit (Millipore; catalogue 17-701). 1 × 10^6^ cells were lysed by RIP lysis buffer, immunoprecipitate with EpCAM antibody or lg G antibody with protein A/G magnetic beads overnight. The immobilized magnetic beads bound complexes were collected and then extract RNA. PCR was performed with primers specific for TINCR promoter (Supplementary Table S2).

### *In vitro* transcription of biotinylated LncRNA-TINCR and RNA pull down

To synthesize TINCR and anti-TINCR transcripts, RT-PCR was conducted using forward primers containing the T7 RNA polymerase promoter sequence. PCR products were purified according to the protocol of Biomiga DNA gel/PCR Extraction Kit (catalogue DC3511-01). TINCR and anti-TINCR were biotinylated according to the protocols of T7 RNA polymerase kit (Beyotime Biotechnology, catalogue D7069) and Pierce^™^ RNA 3′ End Desthiobiotinylation Kit (Thermoscientific catalogue 20163).

RNA pull down assay was conducted according to the instruction of Pierce^™^ Magnetic RNA-Protein Pull-Down Kit (Thermoscientific catalogue 20164). Briefly, labeled TINCR or anti-TINCR was bound to streptavidin magnetic beads, and then incubated the RNA with cell lysate. RNA binding protein complexes were washed and eluted. Western blotting was performed with specific antibody for EpCAM.

### Tumorigenesis and metastasis assay *in vivo*

Four to 5 weeks old athymic nude mice were obtained from the Central Laboratory of Animal Science, Southern Medical University. All experimental procedures involving animals were performed in accordance with animal protocols approved by the Animal Care and Use Committee of Southern Medical University. A total of 2 × 10^6^ HCT116-NC or HCT116-shTINCR cells per animal were subcutaneously injected into the BALB/c nude mice respectively. The size of the tumors was measured by a caliper as length × width^2^ × 1/2 every 2 days. A total of 2 × 10^6^ HCT116-NC or HCT116-shTINCR cells per animal were also injected into the tail veins of the mice, which were sacrificed 6 weeks after injection. The lungs and livers were removed and photographed, and visible tumors on the lungs and livers surface were counted. For histology and immunostaining experiments, subcutaneous tumours, lungs, livers, and the other visceral organs were excised, fixed in formalin, paraffin embedded in paraffin. Sections from the tumours and visceral organs were stained with hematoxylin and eosin (H & E) for general histology, or immunostained with specific antibodies (anti-Ki67 for proliferation) [38].

### Statistical analysis

Statistical analysis was carried out using SPSS 19.0 to assess the differences between experimental groups. Quantitative data were presented as mean ± SD. One-way ANOVA or the independent samples *t* test were used for comparisons independent experimental groups. The spearman correlation coefficient was used to assess the degree of the linear relationship between the expression level of TINCR and EpCAM in colorectal cancer tissues. One asterisk, two asterisks, and three asterisks implied *p* < 0.05, *p* < 0.01, and *p* < 0.001, respectively.

## SUPPLEMENTARY MATERIALS FIGURES AND TABLES


