# Supplementary material for: Loss of TINCR expression promotes proliferation, metastasis through activating EpCAM cleavage in colorectal cancer
Source: Oncotarget. 2016 Mar 17;7(16):22639–49. doi: 10.18632/oncotarget.8141 (PMC5008388; doi:10.18632/oncotarget.8141)
Supplement: Supplementary file 1 [file oncotarget-07-22639-s001.pdf]

Loss of TINCR expression promotes proliferation, metastasis through activating EpCAM cleavage in colorectal cancer

Supplementary Materials

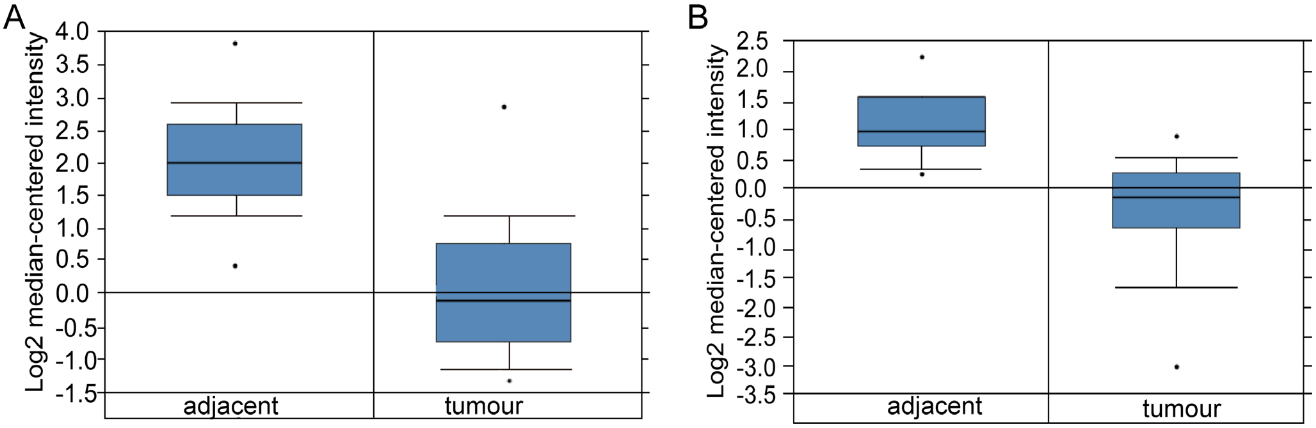

Supplementary Figure S1: The expression of TINCR in Gaedcke's (A) and Hong's (B) research, data from onconmine database.

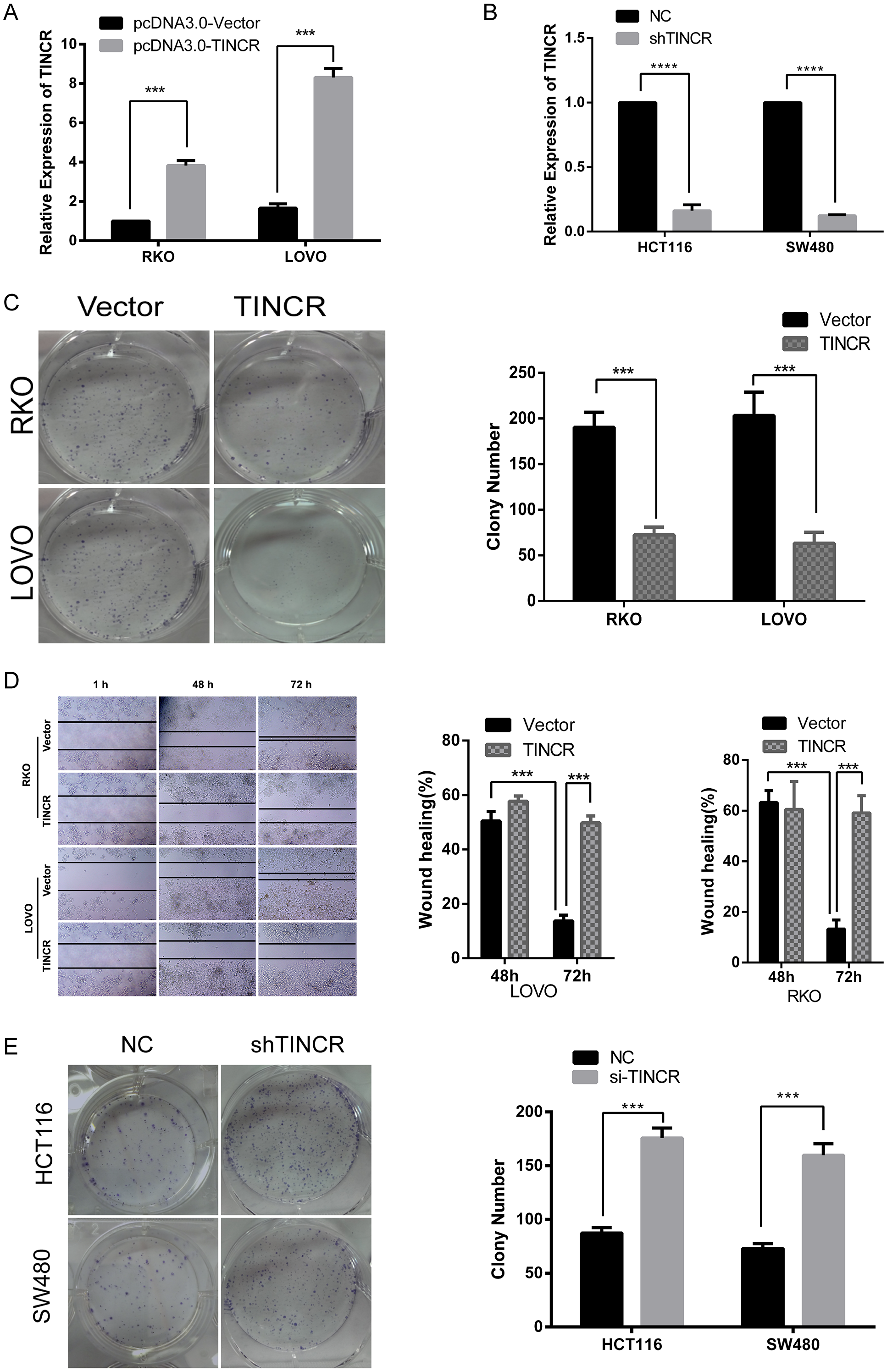

Supplementary Figure S2: TINCR inhibits CRC cells proliferation, migration and metastasis. (**A**, **B**) Construction of HCT116/sh-TINCR, SW480/sh-TINCR cell lines (A), and RKO/oe-TINCR, LOVO/oe-TINCR cell lines (B). Shown data are mean ± SD from 3 independent experiment. (**C**) Colony formation assay were used to analysis cell proliferation in RKO and LoVo cells after oe-TINCR. Shown data are mean ± SD from 3 independent experiment. (**D**) Wound healing assay were used to analysis the effect of oe-TINCR on cell migration in RKO and LoVo cells. Shown data are mean ± SD from 3 independent experiment. (**E**) Colony formation assay were used to analysis cell proliferation in HCT116 and SW480 cells after sh-TINCR. Shown data are mean ± SD from 3 independent experiment.

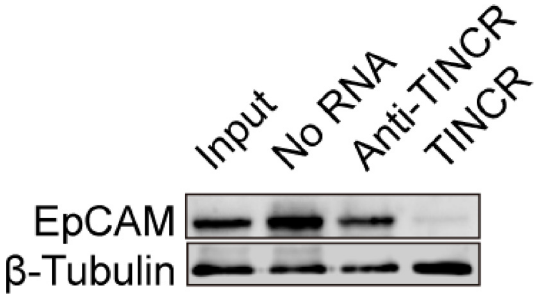

Supplementary Figure S3: RNA pull down assay. Western blotting analysis the EpCAM in the supernatant after incubation with the magnetic bead which was binding to the labeled RNA.

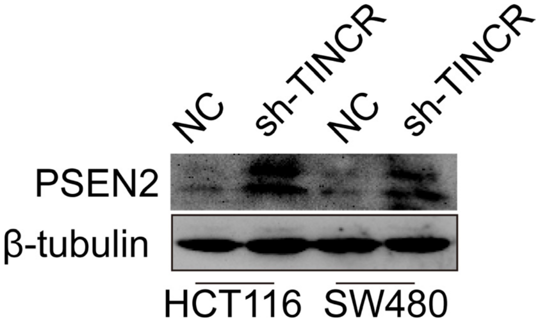

Supplementary Figure S4: Overexpression of psen2 in HCT116/sh-TINCR and SW480/sh-TINCR cell lines.

>TINCR promoter sequence, 1437bp upstream of TINCR transcriptive sequence
CCTGCACCCAGCCCTTCTCTTTTTTTCCTTCTCTTCCTCTCTCTCTCTCTTTCTTTCTTT
CATTCTTTCTTTCCTCTTCTCTTTCTCTCTCTCTCCCTCTTTCTTTCTTTCTTTCTTTCT
TTTTTTTTTTTTTTTTTGAGACGGAGTCTCGCTGTGTCTCCCAGGCTGGAATGCAGTGGC
GCACGATCTCGGCTCACTGCAAGCTCCGCCTCCCAGGTTCACGCCATTCTCTTGCCTCAG
CCTCCGGAGTAGCTGGGACTACAGGCGCCCGCCACCACGCCCGGCTAATTTTTTGTATTT
TTGGTAGAGACGGGGGGGTTTCACCGCGTTAGCCAGGATGGTCTCGATCTCCTGACCTTG
TGATCTGCCCACCTGGGCCTCCCAAAGTACTGGGATTACAGGCGTGAGCCACCACACCCG
GCCATTTTTTTTTTTTTTTTTTTTTTTTTTTTTTTTGCGATAAGTCTCGCTCTGTGGCTC
AGGCTGGAGTGCAGTGGCACAATCTCGGCTCACTGCAACCTCTGTCTGCGGGTTCAAGCG
ATTCTTCTGCCTCAGCCTCCTGAGTAGCTGGAACTACAGGCATGTGCCACCACGCCTGGC
TAATTTTTGTACTTATAGTAGAGACGGGGTTTCACCATGTTGGCCAGGTTGGTCTCGAAC
TCCTGACCTCATGTGATCCGCCCACCTCAGTCTCCCAAAGTGCTGGGATTCCAGGCATGA
GCCACCGCACCTGGCCTAAAAGTAAATTTTTTTTTTAATGTAGGCTGAGCGTGGTGGCTG
AGGGTTATAATCCCACCGCTTTGGGAGGCCAAGGCGGAGAATCACTTGACGTCAGGAGTT
CAAAACCAGCCTGGGCAACACGGCCAGGCCCCATCTCTACAAAAACTACAAAAGTGAGCT
GGGCTTGGTGGCAGCGCCTGTAGTCCCAGCTACTTGAGAGGCTGAGGCGGGAGGATCGCT
TGAGCCCGGGAGGTCAAGGCTGCAACGAACCATGATTGCGCCACTGCGCTCCAGCCTGTC
CTTAAAAAGATCAAATAAAGTGGCCTCCCAGCCTCCCTCCCTGGAGCCTCCACCATGTAA
CCCCCTGGACATTCGCGCGTCTCCTTGGCTGGGATGACCTCGCTGATGGCTCTGTGCTGA
GTGGCCACTCCGCCTCTAGACCCCCGGGCGCACTCTGGGGCCAGCAGTGGGGAGCGGACG
CCTGAGGGGACCGTGGCACCCGCCCCGCCCCGCCGGGCCGGGCCTGCCCACTGCGTGCCG
GGCGTGTCCGGGCGGGCGGAGCGCGGGCGCGGCGGGGGCGGGCGGCCAGGCTAGTCGGGC
GGGTGCGCGGGGCGCTCGGGGCCCGGGGCCAGAGCTGGAGCCGGAGCCGGGCGGGCGCCA
TGGAGGGGCTGCGGCGGGGGCTGTCGCGCTGGAAGCGCTACCACATCAAGGTGCACC

Supplementary Figure S5: The promoter of TINCR.

JARSPAR

Model ID	Model name	Score	Relative score	Start	End	Strand	predicted site sequence
MA0079.3	SP1	14.675	0.96576898177008	1222	1232	1	GCCCCGCCCCG

PROMO

```
Sequence name; Factor name; Start position; End position; Dissimilarity; String; RE equally; RE query
TINCR PROMOTER; Sp1 [T00753]; 1224; 1229; 0.000000; CCGCCC; 0.35083; 0.93622;
TINCR PROMOTER; Sp1 [T00752]; 1221; 1230; 0.000000; GCCCCGCCCC; 0.00548; 0.02440;
TINCR PROMOTER; Sp1 [T00754]; 1221; 1230; 0.000000; GCCCCGCCCC; 0.00137; 0.00709;
TINCR PROMOTER; Sp1 [T00759]; 1223; 1231; 0.175552; CCCGCCCCG; 0.02193; 0.08217;
TINCR PROMOTER; Sp1 [T00755]; 1222; 1230; 0.000000; CCCCGCCCC; 0.00548; 0.02440;
```

Supplementary Figure S6: Bioinformatics analysis the transcriptive factors binding to the TINCR promoter.

Supplementary Table S1: Primers used for plasmid construction

Plasmid	Sense (5′–3′)	Antisense (5′–3′)
pcDNA3.0-TINCR	CCCAAGCTTGGTCTGGGCTCCCAGGTGGACC	AAATATGCGGCCGCTTGTTTTCAAAGATGTAATCTTT
pcDNA3.0-antiTINCR	CCCAAGCTTTTGTTTTCAAAGATGTAATCTTT	AAATATGCGGCCGCGGTCTGGGCTCCCAGGTGGACC
pcDNA3.0-sp1	CCCAAGCTTATGAGCGACCAAGATCACTCCA	CCGGAATTCTCAGAAGCCATTGCCACTGATA
pcDAN3.0-c-myc	CCCAAGCTTCTGGATTTTTTTCGGGTAGTGGA	CCGGAATTCTTACGCACAAGAGTTCCGTAGC
pcDNA3.0-psen2	CGGGGTACCATGCTCACATTCATGGCCTCTG	CCGGAATTCTCAGATGTAGAGCTGATGGGAGGC
pGL3-wt,pgl3-mutation	CGGGGTACCCCTGCACCCAGCCCTTCTCTTT	CCCAAGCTTGGTGCACCTTGATGTGGTAGCGCTT

Supplementary Table S2: Primers used for qRT-PCR

Genes	Sense (5′–3′)	Antisense (5′–3′)
TINCR	TGTGGCCCAAACTCAGGGATACAT	AGATGACAGTGGCTGGAGTTGTCA
EpCAM	TCTGAGCGAGTGAGAACCTA	AGCACAACAATTCCAGCAAC
GAPDH	CATGTTCGTCATGGGTGTGAACCA	AGTGATGGCATGGACTGTGGTCAT
TINCR promoter	TCCCAGGTTCACGCCATTCT	GCTCACGCCTGTAATCCCAGTA
TINCR RNA IP	CCCTCTGTCCTCATTTCCCTG	CTCGTCCTCCCAAAGTGCTG

Supplementary Table S3: Antibodies used in this article

Antibodies	Usage	Corporation and Catalogs
EpCAM	RIP	abcam (ab187372)
EpICD	IHC WB IP	abgent (AJ1263a)
c-myc	WB	proteintech (10828-1-AP)
sp1	WB CHIP	proteintech (21962-1-AP)
ki-67	IHC	abcam (ab66155)
psen2	WB	abgent (AP20576c)
β-catenin	COIP WB	CST (8480p)
TCF-4	WB	proteintech (22337-1-AP)
β-Tubulin	WB	proteintech (10094-1-AP)
caspase-3	WB	proteintech (19677-1-AP)
cleaved caspase-3	WB	proteintech (25546-1-AP)
caspase-9	WB	proteintech (10380-1-AP)
cleaved caspase-9	WB	proteintech (10380-2-AP)

Supplementary Table S4: Clinicopathologic characteristics of TINCR expression in CRC patients

Clinicopathological variables	*N*	High expression	Low expression		
**All case**	**44**	**22**	**22**	X^2	*p*
**Age (years)**[a]					
≤ 60	19	10	9	0.093	0.716
> 60	25	12	13		
**Gender**					
Male	23	13	10	0.820	0.365
Female	21	9	12		
**Tumor size (cm)**[b]					
≤ 4.75	21	11	10	0.091	0.763
> 4.75	23	11	12		
**Differentiation**					
Well	10	8	2	8.098	0.017
Middle	23	12	11		
Poor	11	2	9		
**Serosal invasion**					
Yes	24	7	17	9.167	0.002
No	20	15	5		
**Lymph metastasis**					
Yes	25	9	16	4.539	0.033
No	19	13	6		
**TNM classification**					
I–II	20	14	6	5.867	0.015
III–IV	24	8	16		

[a]Grouping of age was performed according to median.
[b]Tumor size was grouped according to median.
